# Rational design of DKK3 structure-based small peptides as antagonists of Wnt signaling pathway and in silico evaluation of their efficiency

**DOI:** 10.1371/journal.pone.0172217

**Published:** 2017-02-24

**Authors:** Mansour Poorebrahim, Solmaz Sadeghi, Hamzeh Rahimi, Morteza Karimipoor, Kayhan Azadmanesh, Mohammad Ali Mazlomi, Ladan Teimoori-Toolabi

**Affiliations:** 1 Department of Medical Biotechnology, School of Advanced Technologies in Medicine, Tehran University of Medical Sciences, Tehran, Iran; 2 Molecular Medicine Department, Pasteur Institute of Iran, Tehran, Iran; 3 Virology Department, Pasteur Institute of Iran, Tehran, Iran; Russian Academy of Medical Sciences, RUSSIAN FEDERATION

## Abstract

Dysregulated Wnt signaling pathway is highly associated with the pathogenesis of several human cancers. Dickkopf proteins (DKKs) are thought to inhibit Wnt signaling pathway through binding to lipoprotein receptor-related protein (LRP) 5/6. In this study, based on the 3-dimensional (3D) structure of DKK3 Cys-rich domain 2 (CRD2), we have designed and developed several peptide inhibitors of Wnt signaling pathway. Modeller 9.15 package was used to predict 3D structure of CRD2 based on the Homology modeling (HM) protocol. After refinement and minimization with GalaxyRefine and NOMAD-REF servers, the quality of selected models was evaluated utilizing VADAR, SAVES and ProSA servers. Molecular docking studies as well as literature-based information revealed two distinct boxes located at CRD2 which are actively involved in the DKK3-LRP5/6 interaction. A peptide library was constructed conducting the backrub sequence tolerance scanning protocol in Rosetta3.5 according to the *DKK3*-*LRP5/6* binding sites. Seven tolerated peptides were chosen and their binding affinity and stability were improved by some logical amino acid substitutions. Molecular dynamics (MD) simulations of peptide-LRP5/6 complexes were carried out using GROMACS package. After evaluation of binding free energies, stability, electrostatic potential and some physicochemical properties utilizing computational approaches, three peptides (PEP-I1, PEP-I3 and PEP-II2) demonstrated desirable features. However, all seven improved peptides could sufficiently block the Wnt-binding site of *LRP6* in silico. In conclusion, we have designed and improved several small peptides based on the *LRP6*-binding site of CRD2 of *DKK3*. These peptides are highly capable of binding to *LRP6* in silico, and may prevent the formation of active Wnt-*LRP6*-Fz complex.

## 1. Introduction

Wnt signaling pathway regulates cell proliferation and embryonic patterning during development [1]. This signaling pathway has an important role in tumorigenesis of several human cancers and is most often deregulated in colorectal cancer (CRC) facilitating malignant development of polyps in the intestinal epithelium [2, 3]. Nowadays, inhibition of Wnt signaling pathway has been emerged as a promising therapeutic strategy in cancer treatment [4]. In vertebrates, Wnt signaling pathway dissemination is inhibited by two classes of extracellular antagonists: the first class which includes certain members of the DKK family, can bind and inhibit the *LRP5/6* component of the Wnt receptor complex; while the members of second class which comprises secreted frizzled related protein (sFRP) family, WIF (Wnt inhibitory factor)-1 and Cerberus, can directly bind to the Wnt proteins or frizzled receptor [5].

While canonical and non-canonical pathway can be activated downstream to wnt signaling pathway, members of DKK family are highly capable of inhibiting canonical Wnt signaling by targeting *LRP5/6*, and therefore, hinder the formation of Wnt receptor complex. Among members of this family, *DKK1* is the most widely studied member and exhibits a high potential of Wnt inhibition [6]. However, there is now compelling evidence suggesting that DKK3 can also be defined as a key inhibitor of Wnt signaling pathway [7]. A crystallographic study has elucidated that DKKs bind to an ectodomain of *LRP5/6* which comprises four tandem β-propeller—EGF-like domain (PE) pairs. Also it was found that there is an overlapping between Wnt3a- and DKK-binding surfaces on the third PE pair of *LRP5/6* [8]. Therefore, targeting this binding site may interfere with the formation of the active tri-complex (*LRP6*-Wnt-Frizzeld) responsible for initiation of Wnt signaling pathway. Additionally, our in silico analyses established that *DKK3*, similar to other DKK family members, can bind to the third PE pair of LRP5/6 through its CRD2. Interestingly, we found that the CRD2 of *DKK3*, here referred to as *DKK3C*, is bound to the LRP5/6 receptor even tighter than the whole structure. Therefore, this ligand may be regarded as a therapeutic candidate in the cancers which are dependent on the activity of Wnt signaling pathway. Because of their small size and some unique physicochemical properties, small peptides have been widely proposed as therapeutic agents in cancer therapy [9, 10]. It has been demonstrated that use of ligand-derived C-terminal and internal peptides interfering with binding cleft of PDZ domain of Dishevelled (Dvl) proteins, key regulators of Wnt signaling pathway, may be an effective therapeutic strategy for inhibiting Wnt signaling pathway [11]. Nevertheless, due to some intrinsic weaknesses, such as poor physicochemical stability, poor solubility, and short circulating plasma half-life, natural peptides are often not efficiently applicable for use as therapeutic agents [12]. Thus, the weaknesses of initial peptides need to be resolved by computational improvement. Most recently, Zhang Y et al. rationally designed and improved several peptide ligands according to the structure-based information of CaMKIIα-MUPP1 PDZ interaction using a computational mutagenesis approach. The optimized peptides had a high binding affinity to MUPP1 PDZ 11 domain and could competitively disrupt the appropriate interaction between two proteins [13].

To the best of our knowledge, there is no published data reporting the rational peptide designing against Wnt signaling pathway based on the three-dimensional (3D) structure of DKK3. In this study, we have designed several potential inhibitory peptides against Wnt signaling pathway based on the LRP5/6-binding site of DKK3C and evaluated their binding energy, stability and other physicochemical properties in silico. In silico analysis showed that the designed peptides are readily bound to the Wnt- and DKK-binding interfaces of LRP6, and may be considered as possible therapeutic modalities for inhibiting Wnt-mediated cancer progression and invasion.

## 2. Results

### 2–1. Evaluation of three-dimensional (3D) structure of *DKK3C*

**Fig 1:**
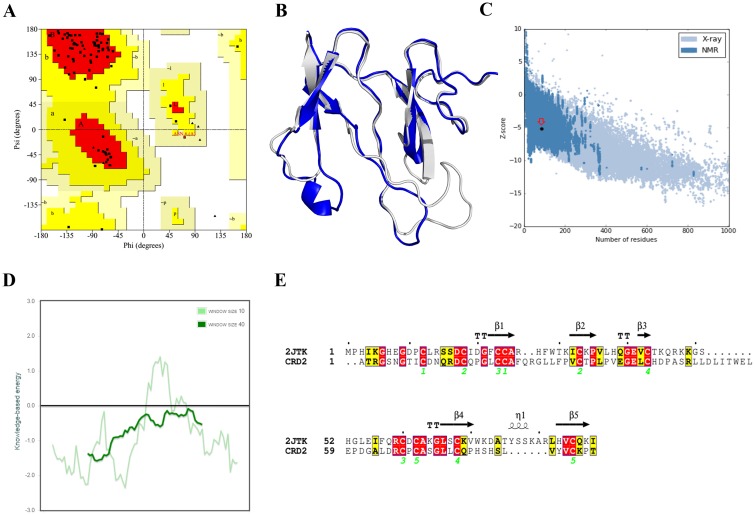
Validation of CRD2 model by several methods. (**A**) Ramachandran plot. The most favored, additionally allowed, generously allowed and disallowed regions are shown in red, yellow, beige and white colors, respectively. (**B**) Structural alignment of *DKK3C* (gray) and 2JTK pdb (blue). (**C**) ProSA Z-score plot of modeled 3D structure of *DKK3C*. The position of this model among experimentally solved protein structures is shown in an open red circle. (**D**) Local model quality by plotting energies as a function of amino acid sequence position. Generally, positive values correspond to problematic parts of the input structure. (**E**) Sequence and secondary structure alignment of *DKK3C* and mouse dkk2 (PDB ID: 2JTK) conducted by ESPript 3.0 (http://espript.ibcp.fr/ESPript/ESPript/).

The 3D structure of *DKK3C* has not yet been determined experimentally, hence we predicted 3D structure of this domain using HM method. The quality of predicted 3D model of *DKK3C* was validated using several methods. Ramachandran plot analysis illustrated that 91%, 8% and 1% of residues were located in the most favored, additionally allowed and generously allowed regions, respectively. There was no residue in the disallowed region of Ramachandran plot (Fig 1A). In order to compare the resulted 3D model of *DKK3C* with the template structure (PDB ID: 2JTK) a structural alignment was performed using TM-align web tool. TM-score of *DKK3C*-2JTK structural alignment was computed to be 0.83 implicating a high accuracy of HM prediction method (two structures with TM-score = 1 have almost the same fold) (Fig 1B). The overall model quality computed by ProSA Z-score was calculated as -5.19 implicating a very high quality of the model when compared with experimentally validated protein structures (Fig 1C). The local model quality was also confirmed using ProSA web tool (Fig 1D). In parallel, a secondary structure alignment was conducted and the result manifested a significant secondary structure compatibility between *DKK3C* and the template model (Fig 1E). The compatibility of 3D-1D structures was evaluated using Verify 3D score. This score assesses the environment of each residue in a model with respect to the high resolution X-ray structures and evaluates the compatibility of the model 3D structure with its sequence by assigning a compatibility score to each residue. Verify 3D was computed as 70.29% implicating a good 3D-1D compatibility of *DKK3C* model (>65% is considered as valid). ModFOLD results uncovered that the predicted model of *DKK3C* was significantly correct (p < 0.001). The global model quality score computed by ModFOLD was 0.7145 implicating the high confident model. The global model quality scores ranged between 0 and 1. Generally, scores less than 0.2 implicate the incorrectly modelled structures and scores greater than 0.4 are indicative of more confident models, which are highly similar to the native structure.

### 2–2. Comparison of *LRP6*-binding interface of CRD2 in *DKK1* and *DKK3*

**Fig 2:**
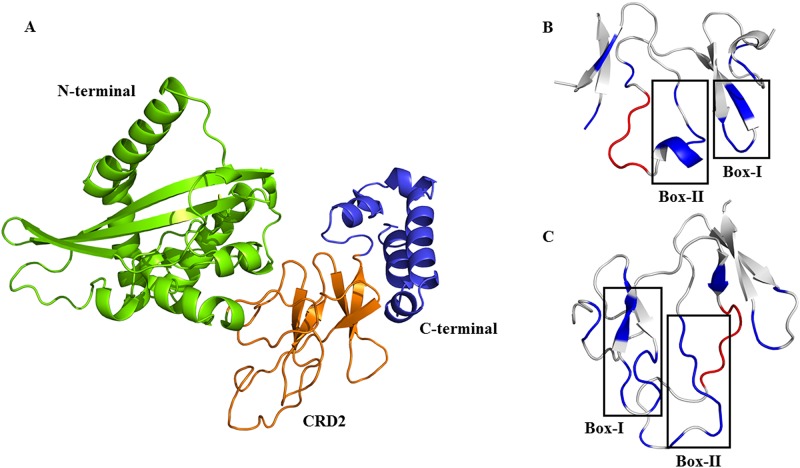
3D structures of whole *DKK3* protein along with CRD2 of *DKK1* and *DKK3*. (**A**) Cartoon representation of 3D structure of *DKK3*; N-terminal, CRD2 and C-terminal regions are shown in green, orange and blue colors, respectively. (**B**) The *LRP6*-binding residues (blue) of CRD2 in *DKK1*. (**C**) The *LRP6* binding residues of *DKK3*. A 6-mer box (red) of positively charged residues in CRD2 of *DKK1* has an important role in *DKK1*-*LRP6* interactions, while this site in CRD2 of *DKK3* (red) has considerably changed with several non-conservative substitutions and has no role in *DKK3C*-*LRP6* interactions.

Comparison of each separately predicted 3D models of whole *DKK3* and its CRD2, after refining and energy minimization steps, showed that *DKK3C* is embedded in the protein comprising from five β-strands along with several coils. Despite other DKK members, there is a region consisting of about 60 residues at the C-terminal of *DKK3* that makes several small helices. This region may prevent the favorable binding of *DKK3C* to *LRP6* (Fig 2A). As mentioned above, molecular docking studies showed that the binding affinity of *DKK3C*-*LRP6* (Δ*G*_*interaction*_ = -14.6 (kcal mol^-1^)) was remarkably higher than whole *DKK3*-*LRP6* complex (Δ*G*_*interaction*_ = -13.8 (kcal mol^-1^)). Although there was a substantial overlap between the *LRP6*-binding sites of *DKK1* and *DKK3*, it was realized that a 7-mer box of *DKK1* which was implicated in the *DKK1*-*LRP6* interaction had no role in the binding of *DKK3C* to *LRP6* (Fig 2B and 2C). This may explain why the binding affinity of *DKK1C* to *LRP6* complex was higher than *DKK3C*-*LRP6* complex. According to the molecular docking results as well as literature-based information, two 11-mer boxes of *DKK3C* (residues 222–232 and 254–264) were chosen for peptide designing. Due to forming more H-bonds and non-bonded connections with *LRP6*, these two boxes could bind to the third PE pair of *LRP6* even stronger than the corresponding *LRP6*-binding sites of *DKK1*. Further confirmation was achieved by using Meta Pocket 2.0 (MPK2) web tool [14]. This server utilizes a consensus method in which the binding sites are predicted by eight different methods including LIGSITECS, PASS, Q-Site Finder, SURFNET, Fpocket, GHECOM, Con-Cavity and POCASA. Indeed, several tools were applied to improve the accuracy of identification of putative binding pockets. Taken together, it was found that some specific residues of each mentioned box were actively involved in the *DKK3C*-*LRP6* interactions. Therefore, these residues were not altered during the steps of affinity maturation and stability enhancement.

### 2-3. Peptide library screening

**Fig 3:**
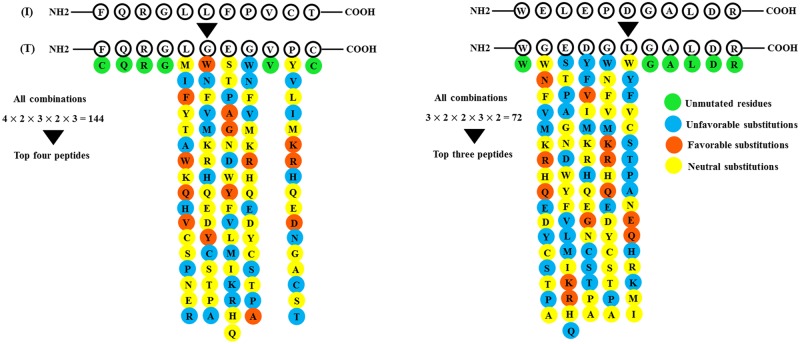
A schematic representation of BoxI (Left) and BoxII (Right) peptide optimization. The initial peptides (I) were tolerated (T) using Backrub and sequence tolerance protocols conducted by Rosetta package. Each position of peptide was substituted with other residues (substitutions with BLOSUM62 score -4 were omitted to avoid deleterious substitutions). The blue, orange and yellow colors indicate unfavorable (Δ*G*_*interaction*_ of resulted peptide > Δ*G*_*interaction*_ of input peptide), favorable (Δ*G*_*interaction*_ of resulted peptide < Δ*G*_*interaction*_ of input peptide) and neutral substitutions (Δ*G*_*interaction*_ of resulted peptide ≊ Δ*G*_*interaction*_ of input peptide), respectively, and the green color represents non-mutated residues. Substitutions that caused a <100 change in the value of interaction weighted score (calculated by ClusPro) were considered as neutral. All possible combinations of favorable substitutions were generated and the best peptides were selected among them.

Based on molecular docking studies as well as literature-based data, two distinct boxes, located at positions 222–232 and 254–264 of *DKK3*, were chosen for generation of peptide libraries. Since, the residues 222, 226–229, 231–232 and 255–259 had a weak binding affinity to *LRP6*, they were substituted with favorable residues to improve the LRP-binding affinity of *DKK3C*. A peptide library was constructed for each box separately. After analysis of constructed libraries using R package and further affinity maturation steps, seven peptides were chosen (four peptides from Box-I and three peptides from Box-II) as the high-scored peptides in case of tolerance, affinity and physicochemical properties. The binding affinity and stability of selected peptides were improved using several repetitions of amino acid substitution, checking binding affinity, stability and other intended features. Only very few mutations on the 11th residue of tolerated peptides were favorable for *LRP6* binding (Δ*G*_*interaction*_ < Δ*G*_*interaction*_ of input peptide). Fig 3 illustrates a schematic representation of the peptide optimization steps.

**Fig 4:**
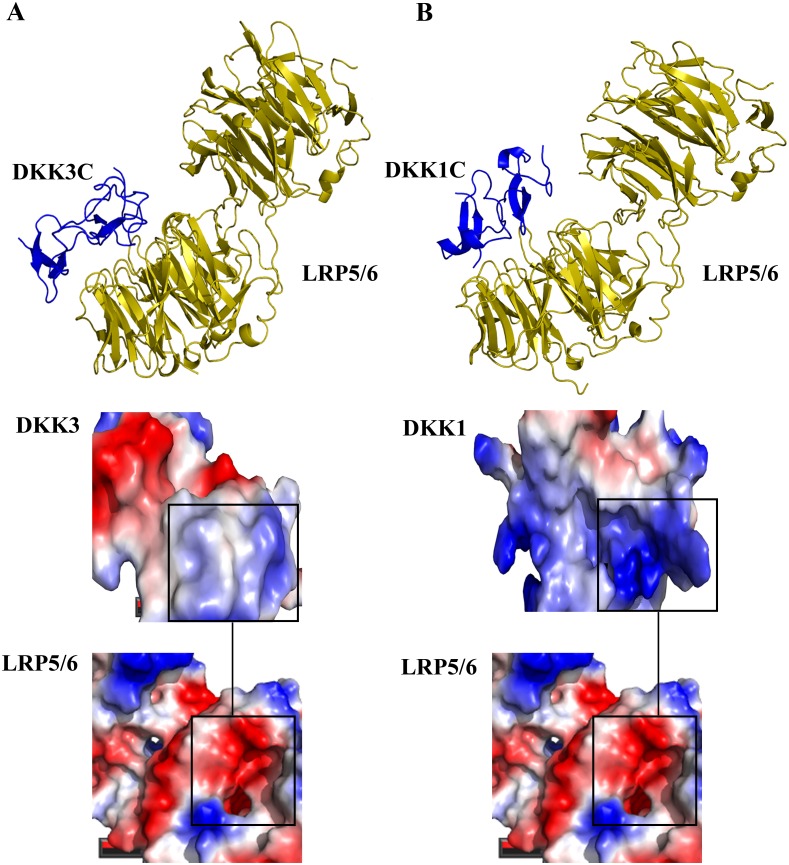
Binding model and surface potentials of *DKK1*/3 and *LRP6*. (**A**) *DKK3C*-*LRP6* and (**B**) *DKK1*-*LRP6* complexes. *DKK1*-*LRP6* complex was obtained from PDB (ID: 3S2K), while *DKK3C*-*LRP6* is the result of ClusPro docking server. The surface potential of binding sites (open circles) calculated by PyMOL software are shown in the bottom of figure. Blue, red and gray colors infer the positive, negative and hydrophobic regions, respectively.

Furthermore, calculation of the surface potential of the *LRP6*-binding interface exhibited that *DKK1* and *DKK3* are mainly bound to a cavity of *LRP6*, a negatively charged site, through a hydrophobic pocket with a high positive surface potential (Fig 4). Consequently, the positive charge and hydrophobicity parameters were considered as important criteria for selecting desired peptides.

Final selected peptides were examined for water solubility and aggregation hot spots. Fortunately, only one of the peptides, named PEP-I4, had poor water solubility and an aggregation hot spot. The details of various calculated properties for each peptide are tabulated in Table 1.

**Table 1:** Binding affinity energy, instability index and physicochemical properties of seven selected peptides.

Peptide Name	Peptide Sequence	Binding Energy	Instability Index*	GRAVY**	Net Charge at PH 7	Water Solubility	Aggregation Hot Spot
**Box-I**							
Initial	FQRGLLFPVCT	-822.2	66.03	0.836	0.9	Poor	No
PEP-I1	CQRGVWARVRC	-1340.2	-19.15	-0.282	2.9	Good	No
PEP-I2	CQRGWYGRVKC	-1159.9	-4.60	-0.927	2.9	Good	No
PEP-I3	CQRGQWYRVDC	-1217.3	-34.52	-1.173	0.9	Good	No
PEP-I4	CQRGFWGAVRC	-1133.1	-22.63	-0.036	1.9	Poor	Yes
Box-II							
Initial	WELEPDGALDR	-733.6	5.17	-1.091	-3	Good	No
PEP-II1	WRKVQEGALDR	-1057.7	4.31	-1.355	1.0	Good	No
PEP-II2	WQKGKQGALDR	-1295.7	0.61	-1.718	2.0	Good	No
PEP-II-3	WNRGRQGALDR	-1143.3	15.53	-1.827	2.0	Good	No

* Instability index value <40 indicates that the peptide is stable.

** The positive and negative GRAVY measures infer the hydrophilicity and hydrophobicity, respectively

### 2–4. The peptide-*LRP6* complexes are highly stable and folded during MD simulation

**Fig 5:**
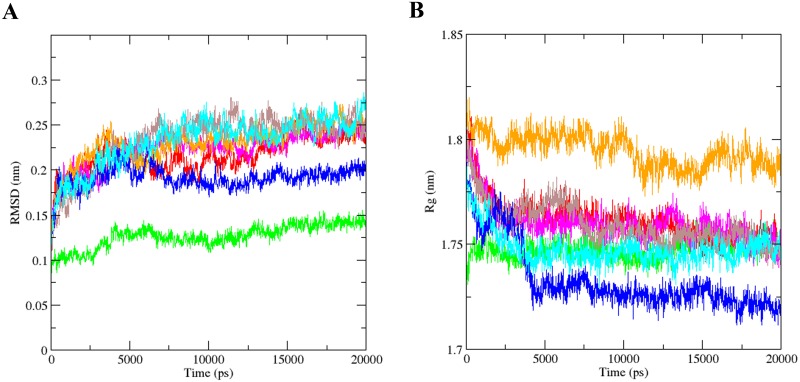
RMSD and Rg plots of peptide-*LRP6* complexes after 20 ns MD simulations. (**A**) RMSD of the backbone atoms and (**B**) Rg plots of PEPI1-*LRP6* (green), PEPI2-*LRP6* (red), PEPI3-*LRP6* (magenta), PEPI4-*LRP6* (orange), PEPII1-*LRP6* (brown), PEPII2-*LRP6* (blue) and PEPII3-*LRP6* (cyan) complexes as a function of time.

The dynamics of the peptide-LRP5/6 complexes were evaluated by comparing the backbone root mean square deviation (RMSD) plots during simulations. RMSD can accurately show a quantitative expression of the conformational changes of peptide-LRP5/6 complexes during simulations. Protein backbone RMSD plots of peptide-LRP5/6 complexes are depicted in Fig 5A. The PEPI1-LRP5/6 and PEPII2-LRP5/6 complexes were stabilized at earlier time as compared to other peptide-LRP5/6 complexes. However, other peptide-LRP5/6 complexes were also stabilized after approximately 5 ns simulations. The lowest RMSD values were seen in PEPI1-LRP5/6 (~0.15 nm) and PEPII2-LRP5/6 (~0.2 nm) complexes, while the RMSD values of other peptide-LRP5/6 complexes were increased until ~0.25 nm and stayed around this value for a period of approximately 10 ns simulations. In parallel, radius of gyration (Rg) values of each peptide-protein complex were also plotted to determine the folded state of each complex during 20 ns MD simulations. Except PEPI4-LRP5/6, other peptide-LRP5/6 complexes were highly compacted and folded during simulations (Fig 5B).

### 2–5. Structural fluctuations and surface accessibility of *LRP6* binding site

**Fig 6:**
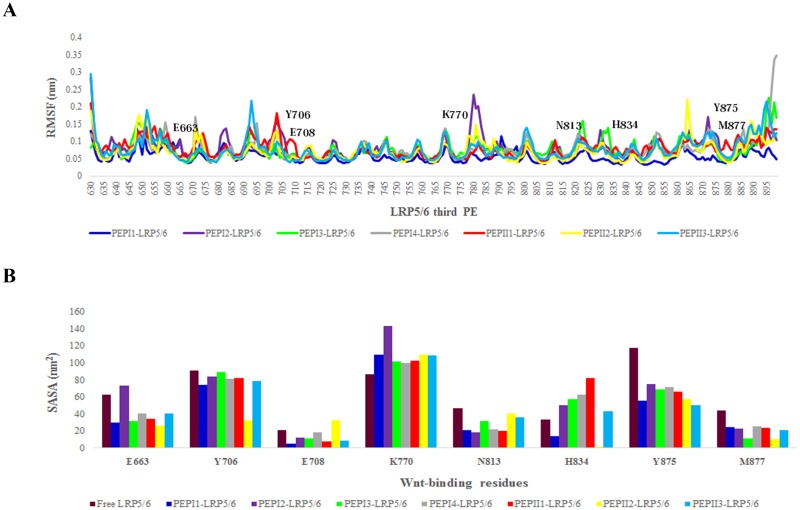
Evaluation of structural fluctuations and surface accessibility. (**A**) The RMSF of each peptide-*LRP6* complex as a function of the residue number in the *LRP6* protein. (**B**) The SASA values for Wnt-binding residues of *LRP6* with or without each designed peptides.

To further assess the structural fluctuations of the peptide-*LRP6* complexes, we calculated the root mean square fluctuations (RMSF) from the trajectories of each peptide-*LRP6* simulations. Focusing on the Wnt-binding residues of *LRP6*, the results disclosed that the mobility of these residues was reduced upon peptide binding, specifically by PEP-I1 and PEP-II2. Indeed, the Wnt-binding site of *LRP6* became more rigid and tended to be less flexible after peptide binding. Considering the different values of RMSF, *LRP6* exhibited a different mobility when it bound to the peptides. Very low fluctuations of Wnt- and DKK-binding residues of *LRP6* is most likely due to high stable conformation of peptide-*LRP6* complexes. PEPI1-*LRP6* and PEPII2-*LRP6* complexes illustrated the lowest structural fluctuations throughout MD simulations (Fig 6A). In parallel, the solvent accessibility surface area (SASA) of each Wnt-binding residue was calculated from the trajectories of final 10 ns peptide-*LRP6* simulations. The SASA of Wnt-binding residues in the peptide-*LRP6* complexes were substantially lower than free *LRP6* (except for K770 residue). This suggests that these residues are probably buried upon binding of designed peptides to *LRP6*. The surface accessibility of H834, a key residue of *LRP6* which is involved in binding to Wnt proteins, was significantly decreased upon binding of PEP-I1 and PEP-II2 to *LRP6*. The average of SASA for Wnt-binding site of each complex was calculated as: free *LRP6* (63.04 nm^2^), PEPI1-*LRP6* (41.61 nm^2^), PEPI2-*LRP6* (60.07 nm^2^), PEPI3-*LRP6* (50.63 nm^2^), PEPI4-*LRP6* (52.60 nm^2^), PEPII1-*LRP6* (52.54 nm^2^), PEPII2-*LRP6* (39.04 nm^2^) and PEPII3-*LRP6* (48.54 nm^2^). Fig 6B represents the SASA values of Wnt-binding residues for each simulated peptide-*LRP6* complex.

### 2–6. The peptides in complex with *LRP6* produced high positive electrostatic potential and H-bonds

**Fig 7:**
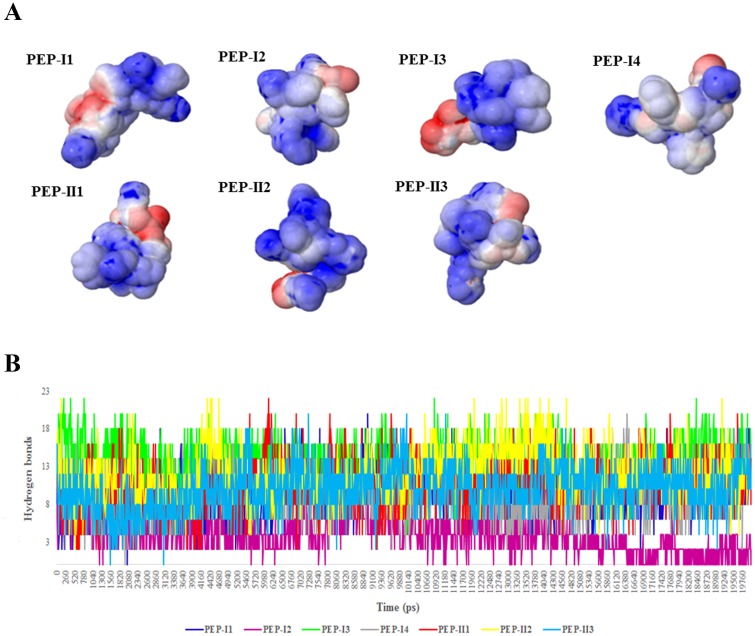
Calculation of electrostatic potentials around the peptides and H-bond numbers of peptide-*LRP6* complexes. (**A**) Electrostatic potentials around the peptides upon binding to *LRP6*. Hydrophobic, positive and negative potentials are shown in gray, blue and red colors, respectively. The highest level of blue and red colors is observed in regions with electrostatic potentials ≥2 and ≤-2, respectively. (**B**) The formation of H-bonds between the peptides and *LRP6* during the production phases of MD simulation.

Electrostatic potential changes upon binding of the peptides were calculated by using normal mode analysis performed by PBEQ-Solver. The average conformation of the peptides was extracted from the last 10 ns of each peptide-*LRP6* simulations. Expectedly, we found a high positive potential around the peptides when bound to the *LRP6* (Fig 7A). Arginine and lysine residues contributed to the high-volume positive coulomb cage bulb around the peptides. While, the negative lobes were attributed to the presence of negatively charged residues, aspartate and glutamate, in the peptide sequence. In addition, the formations of H-bonds between the peptides and *LRP6* during MD simulations was identified. The number of peptide-protein H-bonds in PEPI3-*LRP6* and PEPII2-*LRP6* complexes were obviously higher than other peptides (Fig 7B).

Calculation of several components of peptide-*LRP6* binding free energies obtained from the last snapshots of the MD trajectories showed a strong electrostatic interaction between the peptides and *LRP6*. The net sum of electrostatic interactions between the PEP-I1 and *LRP6* (‹Δ*E*_*electrostatic*_› = -349.13 kcal/mol) was more favorable than other peptide-*LRP6* complexes. The obtained results from energetic analysis of 31 equally spaced snapshots taken from each peptide-*LRP6* MD simulations are summarized in Table 2. Owing to the same structures of each peptide in the non-bound and bound states, the internal component of the ‹Δ*E*_*MM*_›, ‹Δ*E*_*internal*_›, has zero contribution to the binding free energy (‹Δ*E*_*internal*_› = 0) [15].

**Table 2:** Evaluation of peptide-*LRP6* free energies, contacts and interacting surface.

Interaction Free Energy Components (kcal/mol^-1^)							
Energy	PEP-I1	PEP-I2	PEP-I3	PEP-I4	PEP-II1	PEP-II2	PEP-II3
**ClusPro**	-1340.2	-1159.9	-1217.3	-1133.1	-1057.7	-1295.7	-1143.3
**HADDOCK**	-146.4±4.3	-124.8±2.7	-133.8±2.7	-129.9±4.9	-127.9±5.0	-143.7±2.9	-138.1±4.0
**Δ*G*_*interaction*_**	-8.0	-6.8	-7.0	-6.3	-7.0	-8.3	-8.1
**Δ*E*_*electrostatic*_**	-349.13±16.08	-180.47±8.34	-174.23±7.43	-192.49±10.31	-138.61±6.43	-163.08±6.98	-233.28±12.1
**Δ*E*_*VdW*_**	-52.73±9.12	-87.26±14.18	-116.32±18.93	-9.05±2.2	-33.02±4.81	-79.69±8.38	-33.22±4.72
**Δ*G*_*solvation*_**	-373.90 ±21.43	-398.68 ±23.1	-324.21 ±18.6	-318.32 ±14.21	-318.08 ±13.98	-496.38 ±24.69	-285.25 ±12.43
**Δ*E*_*MM*_**	-401.86	-267.73	-290.55	-201.54	-171.63	-242.77	-266.50
***K*_*d*_*(M)***	6.8e-06	1.1e-05	7.5e-06	4.4e-06	1.4e-06	8.2e-07	1.1e-06
**Number of Interfacial Contacts (ICs)**							
**ICs ch-ch**	4	4	6	2	5	5	5
**ICs ch-po**	5	4	2	2	7	7	3
**ICs ch-ap**	16	14	15	11	11	15	13
**ICs po-po**	2	2	2	2	2	1	2
**ICs po-ap**	4	2	4	7	4	6	4
**ICs ap-ap**	13	15	13	16	15	18	16
**Non Interacting Surface (NIS) per property**							
**%NIS ch**	27.54	28.99	28.17	30.54	29.52	30.32	29.80
**%NIS ap**	38.42	38.65	39.44	36.95	38.10	33.84	35.86

*K*_*d*_: Dissociation constant

ch: charge, po: polar, ap: apolar

### 2–7. All peptides could efficiently occupy the Wnt-binding site of *LRP6*

As mentioned before, there is an overlap between the Wnt- and DKK-binding sites of *LRP6*. The putative residues of *LRP6* which are involved in the binding of *LRP6* to Wnt ligands include K770, N813, H834, Y875, M877, E708, E663, K662, Y706 and R1184. These residues are also bound to the members of DKK family. Analysis of peptide-*LRP6* using Ligplot (Dimplot) program revealed that the peptides can readily accommodate on Wnt-binding site of *LRP6*. However, only PEP-I1, PEP-I4 and PEP-II2 could form hydrogen bond with H834, the main residue involved in the Wnt-*LRP6* interactions. All peptides bound to the *LRP6*’s third PE mainly via non-bonded contacts. More details about peptide-*LRP6* interactions are shown in Table 3.

**Table 3:** Residues of *LRP6* interacting with the peptides as predicted by LIGPLOT. Bold residues: Wnt- and DKK-binding, bold and underline: DKK-binding.

*LRP6* Residues		
Peptide Name	Hydrogen bonds forming AAs	Non-bonded contacts forming AAs
**PEP-I1**	L667, M710, D668, G795, **E708**, N794, **Y706**, **E663**, **H834**, **Y875**, L838, D878, I879, R886	G709, A666, A752, **W767**, **R751**, **I681**, A664, A640, **M877**, **F836**, V876, **W850**, G837, L880, T797, T839, L796
**PEP-I2**	T724, **E708**, **R751**, **Y706**, **N794**, **M877**, **E663**, **W850**	L796, A752, G795, S665, D878, **F836**, **Y875**, **H834**, **L810**
**PEP-I3**	G795, T765, **W767**, **R792**, **R751**, **N794**, **E708**, **Y706**, **I681**, **H834**, **Y875**	**L810**, A752, A793, S665, A664, **M877**, **F836**, **W850**
**PEP-I4**	**Y706**, **E708**, R639, **Y875**, **R792**, **R751**	**M877**, **E663**, **W850**, V876, **F836**, **H834**, **L810**, **W767**,
**PEP-II1**	**R792**, **L810**, **D811**, **W850**, **Y875**, V876, **M877**,	**E708**, **Y706**, **H834**, **F836**, P835, **E663**, R639
**PEP-II2**	**H834**, **W850**, **Y875**, **R751**, **E708**, D878, S665, **R792**	V876, **M877**, **Y706**, **F836**, **N794**, A666, **L810**,
**PEP-II3**	A664, **E663**, S665, **E708**, D878, **R751**, **R792**, **N794**, **L810**	**M877**, A666, **W767**, **F836**, V876,

## 3. Discussion

Although, *DKK3* was not previously thought to have interaction with *LRP6* [16], emerging evidences indicate that this ligand can also bind to *LRP6* via its CRD2 [17]. In another computational study, Fujii and colleagues reported that a unique 7-amino-acid insertion (L249-E255 in human *DKK3*) and P258 reduces the binding affinity of *DKK3C* to its receptor [18]. Consistent with this study, we found that a LRP-binding interface of *DKK1* with several positively-charged residues (K^222^, H^223^, R^224^, R^225^, K^226^) have substituted to non-conserve residues in *DKK3* (D^243^, P^244^, A^245^, S^246^, R^247^), which along with an insertion of a 4-amino-acid insertion (D250-L253 in human DKK3) may result in a weak binding of *DKK3C* to *LRP6*. Accordingly, we found that there are two distinct boxes in the *DKK3C* that are highly capable of binding to the third PE pair of *LRP6*. Therefore, several small peptides were designed based on these boxes of *DKK3C*. MD simulations revealed that the peptides can efficiently bind to Wnt-binding site of LRP6 and presumably block it.

**Fig 8:**
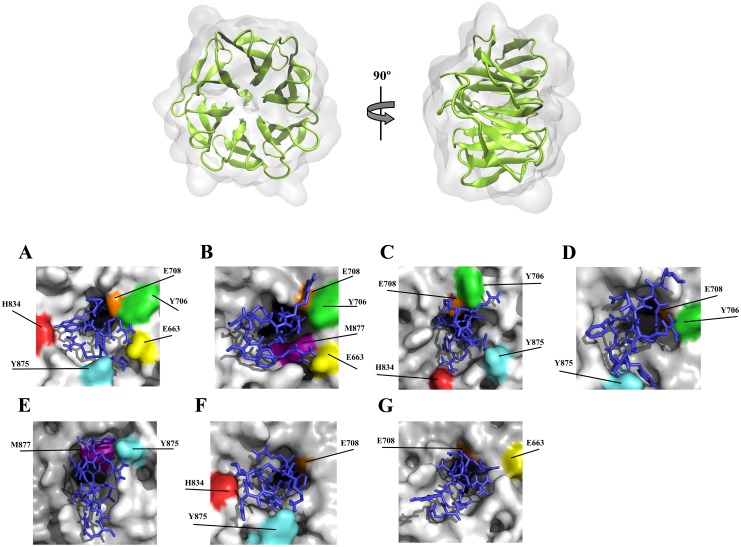
(Up) Representation of third PE pair of *LRP6*. (Down) H-bond interactions between the designed peptides and third PE pair of *LRP6*. (**A**) PEP-I1, (**B**) PEP-I2, (**C**) PEP-I3, (**D**) PEP-I4, (**E**) PEP-II1, (**F**) PEP-II2 and (**G**) PEP-II3 with *LRP6* surface. The common Wnt- and DKK-binding residues of *LRP6* are shown in color. Non-bonded connections have been omitted to simplify the figure.

According to the Chen et al. study, E663, E708, Y875, M877 and H834 residues of *LRP6* are important in its binding to Wnt ligands. However, the impacts of E663 and H834 residues on the Wnt-*LRP6* interaction are significantly higher than other aforementioned residues [8]. The lowest SASA measures for E663 and H834 residues of *LRP6* were observed in PEPI1-*LRP6* (E663 = 30.15 nm^2^, H834 = 13.72 nm^2^) and PEPII1-*LRP6* (E663 = 26.31 nm^2^, H834 = 1.56 nm^2^) complexes compared to free *LRP6* (E663 = 62.4 nm^2^, H834 = 33.85 nm^2^). This suggests that the binding of PEP-I1 and PEP-II2 to *LRP6* may cause a meaningful conformational change in the 3D structure of *LRP6* which results in a reduced availability of E663 and H834 residues for Wnt ligands. The SASA values of other Wnt-binding residues of *LRP6* were also reduced through interaction of the peptides with *LRP6* (Fig 6B). In all cases, except PEP-I4 and PEP-II1 peptides, the peptides were anchored by the hydrogen bonds with at least one of these critical Wnt-binding residues, E663 and H834. Among seven selected peptides, only PEP-I1 could form hydrogen bonds with both E663 and H834 residues simultaneously. The common Wnt- and DKK-binding residues of *LRP6*, which include E663, Y706, E708, K770, N813, H834, Y875 and M877 residues, are probably occupied by the peptides through hydrogen bonds (Fig 8). Studies have shown that I681, S749, R751, W767, G769, R792, N794, L810, D811, D830, F836 and W850 residues of *LRP6* play a pivotal role in the DKK-*LRP6* interactions [19–21]. Our results indicated that all selected peptides could efficiently bind to at least five of these residues simultaneously (Table 3). On the whole, designed peptides may have the ability to selectively interact with Wnt- and DKK-binding residues of LRP6 and result in the prevention of Wnt signaling pathway through blocking Wnt-binding site of *LRP6*.

RMSD and Rg values are widely used for evaluating macromolecules stability and rigidity, respectively [22, 23]. A slight rise of RMSD values of peptide-*LRP6* complexes implicate higher stability of peptide-protein complexes. The highest complex stability was observed in PEPI1-*LRP6* and PEPII2-*LRP6* complexes. Furthermore, Rg values of peptide-protein complexes were decreased during MD simulations. This elucidates that peptide-*LRP6* complexes are highly compacted under physiological conditions. The structural fluctuations of peptide-*LRP6* complexes were also assessed by measuring the RMSF values of each complex during MD simulations. RMSF is an indicator of the macromolecular flexibility and rigidity under thermal equilibrium, and the high fluctuating residues in the protein sequence are highly unstable presumably involved in promoting the protein structure to an unfolded state [24]. Pushie et al. demonstrated that the residues with higher RMSF values are associated with surface-exposed regions involved in protein-protein interactions [25]. PEP-I1, PEP-I3 and PEP-II2 could substantially reduce the RMSF measures of Wnt-binding residues compared to other peptides. The SASA evaluation of Wnt-binding site of *LRP6* also confirmed the reduced accessibility of these residues upon peptide binding (Fig 6).

This is reasonable enough that the surface accessibility of functional residues is crucial for protein function. A reduced SASA value has been known to be associated with a significant reduction of protein activity [26]. Our SASA analysis revealed that Wnt-binding residues of LRP6 tended to be more buried upon peptide-LRP6 interactions. This suggests that the binding of designed peptides to *LRP5/6* may result in the attenuation of Wnt-binding interface of *LRP6* through burying the functional residues involved in Wnt-LRP6 interaction.

Nowadays, electrostatic interactions have been proven to be critical for the catalytic activity and protein—protein recognitions [27]. These energies stabilize the folded state of a protein and play a key role in fold specificity of macromolecules [28, 29]. Cheng et al. have reported that the *DKK1*-binding site of *LRP6* is a hydrophobic patch with some polar residues. They found that the hydrophobic region of this binding site interacts with F205 and W206 residues of *DKK1*. Additionally, several polar interactions including D811 of *LRP6* with R236 of *DKK1*, E708 of *LRP6* with H204 of *DKK1*, R792 of *LRP6* with E232 of *DKK1*, and H834 of *LRP6* with S228 of *DKK1* are actively involved in the maintenance of *LRP6*-*DKK1* complex [21]. Moreover, Lin et al. found that several positively charged residues have a crucial role in the binding of *Mesd* to *LRP6* [30]. These findings underscore that the binding cavities of *LRP6* are largely charged with negatively charged residues. During the peptide binding affinity maturation steps, we considered the high positive surface potential of peptides as an important property for selecting the final peptides. Accordingly, peptides with higher positive surface potentials had a higher binding affinity to the *LRP6* binding site.

### Conclusion

In this study, we have proposed a therapeutic peptide design strategy to interrupt the Wnt-*LRP6* interaction which was based on structural, dynamic and energetic analyses of the *DKK3C*-*LRP6* interaction as well as the previous published data. The designed peptides are highly capable of binding to the Wnt- and DKK-binding sites of *LRP6* in silico, in a selective manner. These peptides, especially PEP-I1 and PEP-II2, could induce a local conformational change in *LRP6* structure leading to an increased buried surface area of Wnt-binding site. The peptide-LRP6 complexes were highly stable and compact, and therefore, could be considered as possible therapeutic agents for hindering Wnt signaling pathway in the cancers which are dependent on function of this signaling pathway. However, the impacts of the selected peptides on Wnt signaling pathway need to be validated experimentally.

## 4. Materials and methods

### 4–1. Modeling, refinement and quantitative evaluation of *DKK3C*

Modeller v9.15 software was used for performing HM using the crystal structure of CRD2 of the mouse dkk2 (PDB ID:2JTK) as the template [31]. The HM method consists of the following steps: (i) template selection; (ii) target template alignment; (iii) model building; and (iv) model evaluation. These steps can be iteratively repeated, until a reasonably accurate model is generated. The sequence identity and similarity of this template with *DKK3C* were defined to be 30% and 73% respectively. Among 10000 generated models, 10 top models were chosen (based on their DOPE score) for further analyses. Evaluation of the models quality was carried out by VADAR, SAVES (https://services.mbi.ucla.edu/SAVES/), ProSA and ModFOLD web tools [32–34]. Parallel checking of model quality was conducted utilizing several tools to enhance the accuracy of validation. Finally, the best model was selected and considered for further analyses. GalaxyRefine and NOMAD-REF servers were used for refining and energy minimizing the selected model [35]. These servers rebuilt the model side-chains and performed overall structure relaxation using MD simulations. The low free-energy conformations were further refined by full-atomic simulations using ModRefiner method [36].

### 4-2. Construction of peptide library

The stability of initial peptide sequences was remarkably low; therefore, a focused peptide library was constructed based on all possible amino acid substitutions. According to the molecular docking studies and literature data mining, some residues were recognized as the key residues involved in *DKK3*-*LRP6* interactions, and therefore, were not manipulated. A peptide library was constructed using Rosetta backrub and sequence tolerance protocols implemented in the Rosetta3.5 software [37]. Using these protocols, a given peptide sequence can be tolerated while still preserving its function at the defined level. The results were analyzed using R package and the tolerated peptide sequence was generated [38].

### 4-3. Optimization of binding affinity and stability of the peptides

First, the initial peptide sequences were tolerated utilizing Rosetta backrub and sequence tolerance protocols. However, the binding affinity of these peptides, both BoxI- and BoxII-derived peptides, to *LRP6* were not satisfactory and needed to be improved. To this end, several steps of affinity maturation were performed by generating some logical substitutions. BLOSUM-62 scoring matrix, one of the most effective matrices for predicting the common amino acid substitutions, was used to generate non-deleterious substitutions [39]. Except the amino acid substitutions with score of -4, any residue was separately substituted with all other amino acids. Nevertheless, the main *LRP6*-*DKK3C* interacting residues which had been predicted by molecular docking studies as well as literature-based information did not change. Following each substitution, the binding affinity, stability and several physicochemical properties of the peptides were evaluated. At each position, the amino acid substitutions that lead to an increase in the binding affinity of the peptides to *LRP6* were distinguished. All possible combinations of favorable substitutions were generated and their binding energy to *LRP6* was calculated again. In this step, MD simulations were not carried out due to the high computational demand. Finally, seven improved peptides (Box-I: four peptides, Box-II: three peptides) were selected for production of MD simulation trajectories.

### 4-4. Evaluation of binding affinity and stability of peptide variants

Any substitution was followed by evaluating peptide binding affinity and stability using ClusPro docking server and ProtParam (http://us.expasy.org/tools/protparam.html) web tool, respectively [40]. In parallel, a molecular docking was separately conducted using HADDOCK server to validate the peptide-*LRP6* interaction mode. This program is a powerful docking tool which uses a data-driven approach supporting for a wide range of experimental data [41]. ClusPro assigns a weighted score for binding energy of a given protein complex. ProtParam calculates the instability index of the proteins and peptides. Instability index is a reliable measure that provides an estimate of the stability of a protein in a test tube by using statistical analysis of 12 unstable and 32 stable proteins. The proteins and peptides with instability index lower than 40 are classified as stable [42]. Prior to docking, the structure of each peptide was predicted by using PEP-FOLD server [43]. The initially docked peptide-protein complexes resulted from ClusPro server were refined using GalaxyRefineComplex web tool (http://galaxy.seoklab.org/cgi-bin/submit.cgi?type=COMPLEX).

### 4-5. Calculation of physicochemical properties of peptides

Several physicochemical properties of the peptides including molecular weight, net charge at pH 7, pI, water solubility, aliphatic index, grand average of hydropathicity (GRAVY) and aggregation hot spots were calculated using ProtParam, PepCalc and Aggrescan tools [44, 45]. It is now widely accepted that a positive GRAVY value infers the hydrophobicity, while a negative GRAVY value indicates the hydrophilicity level of a protein [46]. Considering the binding affinity and stability, the aforementioned parameters were also computed to choose the best three peptides with completely matched properties for in vitro experiments.

### 4-6. MD simulations

In order to assess the conformational changes of the peptides, alone and in complex with *LRP6*, MD simulations were carried out using GROMACS version 5 for a period of 20 ns using the original GROMOS96 force field 43A1 [47]. MD simulation is a very efficient tool that is commonly used in the solid-state physics and materials community for modeling solids and liquids at the atomic level. Single peptides as well as peptide-*LRP6* complexes were solvated in a solvation box using SPC/E water model with 10Å distance between the edges of the box and the peptides. The system was neutralized by replacing solvent molecules with Cl^-^ and Na^+^ ions. Subsequently, the system was relaxed using steepest descent algorithm followed by conjugate gradient algorithm. After several energy minimization steps, the entire system was equilibrated for 100 ps using canonical (NVT) and the isothermal—isobaric (NPT) ensembles. Finally, the equilibrated system was simulated for a period of 20 ns using NPT ensemble to understand the structural dynamics of the peptides undergoing interaction with *LRP6*.

### 4-7. Visual presentations

All protein figures were created using PyMOL and VMD programs [48, 49]. The graphs obtained from MD simulations were mainly plotted using the Grace software (GRACE: http://plasma-gate.weizmann.ac.il/Grace/).

### 4-8. Calculation of the electrostatic potential around the peptides

The electrostatic potential around the selected peptides was calculated for the extracted structures of the peptides derived from peptide-*LRP6* MD simulations. We used the average coordinates of peptide structures extracted from the final 10 ns simulations (10–20 ns) for the electrostatic potentials calculations. The PBEQ-Solver [50] was used for calculating electrostatic potential surface around each peptide by solving the nonlinear Poisson-Boltzmann (PB) equations with a grid spacing of 1 ‎Å [27].

### 4-9. Energetic analysis

To calculate the binding energy of each peptide-*LRP6* complex, an average structure was prepared from the last 10 ns MD simulations. The electrostatic and van der Waals (VdW) energies were calculated for the last snapshot configurations taken from the MD trajectories of peptide–*LRP6* complexes and then averaging the values. Unbound peptides and *LRP6* snapshot configurations were also prepared from the peptide-*LRP6* complex trajectories. The coordinates of 31 snapshots were taken at 100-ps intervals during the last 3 ns simulations of each peptide-*LRP6* complex, where the complexes appeared to acquire a stable configuration, and used for energetic analysis.

#### 4-9-1. Gibbs free energy of the peptide-*LRP6* interaction

The Gibbs free energy change (ΔG) for all selected peptides undergoing the interaction with *LRP6* were calculated from the atomic structures of peptides and *LRP6* receptor. The interaction energy calculations for each peptide-*LRP6* complexes were computed for their average structures extracted from the last 10 ns of MD production phase. PRODIGY web tool was used for measuring ΔG of peptide-*LRP6* interactions as well as their polar and nonpolar contacts [51]. This server computes the number of interfacial contacts (ICs) and percentage of non-interacting surface (NIS) in a protein-protein complex which have been demonstrated to be the key parameters in the protein-protein binding energy prediction [52]. Free energy of interaction was calculated as follow:

ΔGinteraction=−0.09459 ICscharged/charged−0.10007 ICscharged/apolar+0.19577 ICspolar/polar−0.22671 ICspolar/apolar+0.18681 %NISapolar+0.3810 %NIScharged−15.9433

Where, ICs_xxx/yyy_ is the number of Interfacial Contacts found between the interface of the first and the second interactors classified based on the polar/apolar/charged nature of the interacting residues (i.e. ICs_charged/apolar_ is the number of ICs between charged and apolar residues). The contacts between two residues were defined if any of their heavy atom was within a distance of 5.5 Å.

#### 4-9-2. Solvation energy

The solvation energy consists of the electrostatic (Δ*G*_polar, solvation_) and nonpolar (ΔG_apolar, solvation_) contributions. Solvation energies of the peptides were presented as the average values of the energies calculated for the snapshot configurations at the last 3 ns MD simulations. The PBEQ-Solver has been used for calculating the solvation energy of each peptide.

#### 4-9-3. Molecular Mechanics (MM) calculations

The free binding energies of peptide–*LRP6* complexes have been computed with the MM according to over snapshots from MD trajectories. The 31 equally spaced snapshot configurations extracted from the last 3 ns peptide-*LRP6* simulations were used for energy calculations. *‹E*_*MM*_*›* is generally calculated as follow:

〈EMM〉=〈Einternal〉+〈Eelectrostatic〉+〈EVdW〉

Where, the (*E*_*internal*_) value includes bond, angle, and torsional angle energies, (*E*_*electrostatic*_) and (*E*_*VdW*_) values refer to the non-bonded electrostatic and van der Waals interactions of the peptides and proteins. The value *E*_*electrostatic*_ was calculated using APBS [53] as implemented in PyMOL. The *E*_*VdW*_ value was calculated using a standard GROMACS utility with the same force field applied in the MD simulations (No cutoff was defined for the evaluation of non-bonded interactions).
